# Evaluating and forecasting movement patterns of magnetically driven microbeads in complex geometries

**DOI:** 10.1038/s41598-020-65380-8

**Published:** 2020-05-29

**Authors:** Finn Klingbeil, Findan Block, Umer Sajjad, Rasmus B. Holländer, Sughosh Deshpande, Jeffrey McCord

**Affiliations:** Institute for Materials Science, Kiel University, Kaiserstraße 2, D-24143 Kiel, Germany

**Keywords:** Applied physics, Techniques and instrumentation, Lab-on-a-chip

## Abstract

The manipulation of superparamagnetic microbeads for lab-on-a-chip applications relies on the steering of microbeads across an altering stray field landscape on top of soft magnetic parent structures. Using ab initio principles, we show three-dimensional simulations forecasting the controlled movement of microbeads. Simulated aspects of microbead behaviour include the looping and lifting of microbeads around a magnetic circular structure, the flexible bead movement along symmetrically distributed triangular structures, and the dragging of magnetic beads across an array of exchange biased magnetic microstripes. The unidirectional motion of microbeads across a string of oval elements is predicted by simulations and validated experimentally. Each of the simulations matches the experimental results, proving the robustness and accuracy of the applied numerical method. The computer experiments provide details on the particle motion not accessible by experiments. The simulation capabilities prove to be an essential part for the estimation of future lab-on-chip designs.

## Introduction

Downsizing and integrating the functionality of traditional laboratory capabilities onto a microchip is a focus of research on lab-on-a-chip devices^1–8^. The building blocks of these devices are functional elements like sensors or valves connected by microfluidic channels^9^. At the same time the controlled manipulation of magnetic particles has gathered much attention for possible bio-applications, allowing transport, separation, and detection of magnetic micro- or nanoparticles^7,10–12^. Functionalized superparamagnetic microbeads (MB) are widely used in labelling and detection of biomedical species^13–15^.

The movement of MBs on magnetic parent structures has been investigated experimentally using different schemes utilizing structured hard magnets^14,16,17^, soft magnets^7,12,18,19^, or magnetic thin full films as the parent structure^10,20–22^. For soft magnetic structures, a large number of designs such as rings^12,23^, stripes^20,24^, periodic arrays of elements^7,19,25^, and mixed structures^26^ can be employed to generate particle specific paths of motion. In general, the behaviour of the MBs on the magnetic structures is defined by the magnetostatic interaction between the MBs and the magnetic structures over which an external magnetic vector field is applied. By changing the external field and thereby the magnetic microstructure of the employed magnetic structures, motion of the MBs is achieved by changing the correlated potential energy landscape. In these cases, the speed of motion is limited by the hydrodynamic drag and surface friction acting on the MB for a fixed potential energy gradient.

The modelling of MB behaviour by quantitative descriptions of the magnetic forces between the superparamagnetic MBs and the micromagnetic state of the parent structure, as well as the hydrodynamic drag forces, is of great interest for the design of new structures to achieve specific functionalities^12,16,27,28^. Various simulations considered different aspects of MB motion with magnetic patterns. This includes the aspects of MB trapping^29^ and resulting magnetic potentials of the magnetic structures from micromagnetic calculations for the design of microrotors^30^ and magnetic tracks displaying bead size selective MB moving behaviour^31^. Complete MB movements were modelled using semi-analytical approaches for the description of the underlying varying magnetic microstructure^32^. The most complete modelling of MB movement was presented in ref. ^32^ and ref. ^18^, where the spatial and dynamic MB conduct was modelled. Yet, all of the above modelling is restricted to two-dimensional MB movement across the patterned magnetic surfaces.

Here, we predict static and dynamic MB behaviour in a wide range of structures by three-dimensional simulations, facilitating base functionalities of transport and sorting of MBs. We demonstrate the ability to simulate arbitrary structures starting from calculated states of magnetization being computed for all relevant external magnetic field conditions. To model MBs progressing along an array of structures over large distances the possibility of including periodic and moving boundary conditions was implemented. Arbitrary magnetic field sequences are numerically applied. Using the three-dimensional modelling, magnetic potentials in the out-of-plane axis of the structures, which may lead to forces lifting MBs of the ground, are taken into account. A selected variety of different model and application relevant systems is used to validate the accuracy of the calculations.

### Modelling background

For the simulations we assume that the movement of a MB in the *xy*-plane is only influenced by the magnetic force ***F***_**m**_, which origins from a magnetic potential gradient, the hydrodynamic drag force ***F***_**hd**_, depending on the velocity and the radius of the MB, as well as the frictional force ***F***_**f**_ between the MB and substrate. For movement along the *z*-axis perpendicular to the *xy*-plane the gravitational force ***F***_**g**_ must be considered. The total acting force ***F***_**a**_ on the MB is

1 $${{\boldsymbol{F}}}_{{\boldsymbol{a}}}={{\boldsymbol{F}}}_{{\boldsymbol{hd}}}+{{\boldsymbol{F}}}_{{\boldsymbol{m}}}+{{\boldsymbol{F}}}_{{\bf{f}}}\,+\,{{\boldsymbol{F}}}_{{\boldsymbol{g}}}\,.\,$$

In equilibrium, the out-of-plane component of the magnetic force enforces the particle in a two-dimensional plane on top of the substrate, but during dynamic motion, out-of-plane magnetic forces may lift the MB off into damped 3D-motion.

The starting point for the calculation of the magnetic force is the micromagnetic simulation of the magnetic states of the ferromagnetic parent structure. The magnetization configuration of the parent structures ***M***_p_ for all relevant external magnetic field states is calculated using the GPU-based micromagnetic simulation program Mumax^33^. This allows for an exact description of the magnetization state, independent of the system of investigation. It forms the basis for our simulations of MB motion, the relevant aspects of which being described next.

From the magnetic states, the magnetic potential maps of the MB are calculated, assuming the MBs as a magnetic dipole at a height *z*_mb_ of *z*_*m*b_ = *r*_mb_, corresponding to the position along the out-of-plane direction *z* at the radius *r*_*m*b_ of the MB. For the calculations we assume that the uniform MB magnetization ***M***_**mb**_ oriented along the external field ***H***_**ext**_ is proportional to the susceptibility *χ*, with

2 $${{\boldsymbol{M}}}_{{\bf{m}}{\bf{b}}}=\chi {{\boldsymbol{H}}}_{{\bf{e}}{\bf{x}}{\bf{t}}}\,.\,$$

The stray field ***H***_**mb**_ of the MB as a function of the radial distance *r* is then given by

3 $${{\boldsymbol{H}}}_{{\bf{m}}{\bf{b}}}=\frac{1}{4\pi \cdot {r}^{2}}\frac{3r({{\boldsymbol{M}}}_{{\bf{m}}{\bf{b}}}\cdot r)-{{\boldsymbol{M}}}_{{\bf{m}}{\bf{b}}}{r}^{2}}{{r}^{3}}\,.\,$$

The assumption of treating the MBs as magnetic single dipoles with a stray field ***H***_**mb**_ is valid for a homogenous distribution of magnetic material in the (in our case) MB shell. The susceptibility of the MB is fitted by using data from the well-known model structures presented in^18^.

The total magnetic potential *U* is the sum of the magnetic potentials of the MB *U*_mb_ and the parent structure *U*_p_. We calculate both by integrating the scalar product of their magnetization and all other field sources over their total volume *V*, with

$$U={U}_{{\rm{mb}}}+{U}_{{\rm{p}}}$$

4 $$\,=-\,{\mu }_{0}{\int }_{{V}_{{\rm{mb}}}}dV{\prime} {{\boldsymbol{M}}}_{{\bf{m}}{\bf{b}}}\cdot ({{\boldsymbol{H}}}_{{\bf{e}}{\bf{x}}{\bf{t}}}+{{\boldsymbol{H}}}_{{\bf{p}}})-{\mu }_{0}{\int }_{{V}_{p}}dV{\prime} {{\boldsymbol{M}}}_{{\bf{p}}}\cdot ({{\boldsymbol{H}}}_{{\bf{e}}{\bf{x}}{\bf{t}}}+{{\boldsymbol{H}}}_{{\bf{m}}{\bf{b}}}).$$

Using the reciprocal theorem^34^, we eliminate the magnetic stray field of the parent structure ***H***_**p**_ by integrating over the magnetization of the parent structure ***M***_**p**_ and the MBs dipole stray field ***H***_**mb**_. The total magnetic potential *U* is then

5 $$U=-\,{\mu }_{0}{\int }_{{V}_{{\rm{p}}}}dV{\prime} {{\boldsymbol{M}}}_{{\bf{p}}}\cdot ({{\boldsymbol{H}}}_{{\bf{e}}{\bf{x}}{\bf{t}}}+2\cdot {{\boldsymbol{H}}}_{{\bf{m}}{\bf{b}}})+{C}_{0}.$$

Calculations are performed only for the parts of the integral that affect the gradient of *U*, thus the integration constant *C*_0_ is ignored. The potential map is created by using an additional grid with a selectable spatial resolution, where every grid point defines a different position of the MB relative to the parent structure. In general, the runtime for the calculation of the potential can be reduced by averaging the magnetization data over neighbouring cells in the *xy*-plane.

To obtain high temporal resolution, for a given field sequence the local dependence of the potential on the time *t* is then approximated with a Fourier series fit using a standard Fourier series algorithm up to the coefficients of an order *N*, which is selected based on the magnetic field sequence. The Fourier expansion is labelled here as ${{\bf{F}}}_{\,1}^{T}(t)=(1,\,\cos (\omega t),\ldots ,\,\cos (N\omega t))$ and ${{\bf{F}}}_{\,2}^{T}(t)=(0,\,\sin (\omega t),\ldots ,\,\sin (N\omega t))$. The spatial fitting of the potential is performed using a polynomial approach. The potential maps are subdivided into smaller 3 × 3 × 3 or 5 × 5 × 5 submatrices and the spatial dependency depending on the position of the MB ***s***(***x, y, z***) is determined for each submatrix with a polynomial fit using the polynomial expansion ${{\bf{p}}}^{T}({\boldsymbol{s}})=(1,{\boldsymbol{x}},{\boldsymbol{y}},{\boldsymbol{z}},{\boldsymbol{xy}},{\boldsymbol{xz}},{\boldsymbol{yz}},{\boldsymbol{xyz}},{{\boldsymbol{x}}}^{2},{{\boldsymbol{y}}}^{2},{{\boldsymbol{z}}}^{2})$. The local potential energy distribution *U* can be calculated as:

6 $$U(t,{\boldsymbol{s}})={{\bf{F}}}_{\,1}^{T}(t){\mathscr{A}}{\bf{p}}({\boldsymbol{s}})+{{\bf{F}}}_{\,2}^{T}(t){\mathfrak{B}}{\bf{p}}({\boldsymbol{s}})$$

The matrices ${\mathscr{A}}$ and ${\mathfrak{B}}$ contain the numerical coefficients obtained by the Fourier and polynomial fit algorithms. The negative gradient of these fits corresponds to the local magnetic forces

7 $${{\boldsymbol{F}}}_{{\bf{m}}}=-\,\nabla U.$$

For the drag force it is possible to derive the drag by a stokes term from the Basset–Boussinesq–Oseen equation^35^, giving the linear hydrodynamic damping parameter Γ_hd_ assuming no pressure gradient as well as steady forces^18^:

8 $${\Gamma }_{{\rm{hd}}}=6\pi \eta {r}_{{\rm{mb}}}$$

The viscosity of the aqueous medium *η* is calculated based on the temperature behaviour of water^36^. Finally, we take the added effective mass term *m*_eff_, related to the densities of the MB ρ_mb_ and of water ρ_water_ into account:

9 $${m}_{{\rm{eff}}}=\left(\frac{4\pi }{3}\right){r}_{{\rm{mb}}}^{3}\cdot \left({\rho }_{{\rm{mb}}}+\frac{{\rho }_{{\rm{water}}}}{2}\right)$$

The frictional force^37^ is dependent on the friction coefficient F_c_ multiplied by the magnetic force acting in negative z-direction ***F***_**mz**_. It acts opposite to the direction of movement. The friction coefficient F_c_ is set for z = 0 and is zero for all other z-positions.

10 $${{\boldsymbol{F}}}_{{\bf{f}}}=-\,{{\rm{F}}}_{{\rm{c}}}(z)\cdot |{{\boldsymbol{F}}}_{{\bf{m}}{\bf{z}}}|\cdot \frac{\frac{d{\boldsymbol{s}}}{dt}}{|\frac{d{\boldsymbol{s}}}{dt}|}$$

Combining these terms leads to the equation of motion:

11 $${m}_{{\rm{eff}}}\cdot \frac{{{\rm{d}}}^{2}{\boldsymbol{s}}}{d{t}^{2}}=-\,{\Gamma }_{{\rm{hd}}}\cdot \frac{d{\boldsymbol{s}}}{dt}\cdot \left(\begin{array}{c}1+\frac{9{r}_{{\rm{mb}}}}{16({r}_{{\rm{mb}}}+z)}\\ 1+\frac{9{r}_{{\rm{mb}}}}{16({r}_{{\rm{mb}}}+z)}\\ 1+\frac{9{r}_{{\rm{mb}}}}{8({r}_{{\rm{mb}}}+z)}\end{array}\right)+{{\boldsymbol{F}}}_{{\bf{m}}}+{{\boldsymbol{F}}}_{{\bf{f}}}$$

The additional term modifying the stokes drag is a linear approximation dependent on the ratio of the MB radius ${r}_{{\rm{mb}}}$ to the distance of the MB centre from the substrate $({r}_{{\rm{mb}}}+z)$^35^. It describes the increase in drag when the MB is moving close to the substrate.

The equation of motion is iteratively evaluated for time steps of a maximum of 7∙10^−4^ s. using MATLAB’s ode15 solver^38^, determining the *x* and *y* positions and velocities at the solver time, where the numerical solution is closest to a new grid point. We then use the nearest grid point along the trajectory as the centre of the submatrix for the next solver step.

For periodic and extended structures, the magnetization distribution is calculated using periodic boundary conditions^33^. We likewise adapt period boundary conditions for calculating the potential along the periodic directions. This is followed by introducing overlapping submatrices at the borders of the potential maps for fitting and by adjusting the solver output, when the MB moves over a boundary. The periodic boundaries can be in either along one or along both of the in-plane directions.

As long as a MB is in the vicinity of a potential minimum the magnetic forces along the *z* direction lead to a magnetic force pointing downward into the surface of the parent structure. The origin of the z-axis is defined in a height of *r*_mb_ above the substrate surface, corresponding to the height of the centre point of the MBs positioned at the surface. If in our calculations the MB during its movement is forced to positions of relative maxima in addition to the in-plane gradients, a gradient along the *z* direction exists that favours elevation of the MB from the parent structure’s surface. Qualitatively, we have already observed this behaviour for the looping events presented in^18^. In order to perform three-dimensional calculations, the potential map is determined in a range of heights varying from *z*_mb_ = −*r*_mb_/2 to a height *z*_mb-max_ that is greater than the maximum height the MB will reach above the parent structure. We choose the spacing between the different heights comparable to the spacing of the grid points in the *xy* potential maps. In the spatial fit, the potential is then approximated along the *z* direction.

For taking the out-of-plane magnetic forces into account, in the equation of motion we further consider the gravitational force ***F***_***G***_. For three-dimensional calculations, for each solver step the force term in the equation of motion is chosen based on the trajectory of the MB. At the start of each solver step, the acting force for the equation of motion ***F***_***i***_ is obtained, where ***F***_***i***_ includes all force contribution not depending on time derivatives of ***s****.* If the MB is at the ground level and no magnetic force stronger than gravity points out-of-plane upwards, corresponding to ***F***_**i-1**_(*z*) ≤ 0, we solve the time step with the two-dimensional in-plane equation of motion. Otherwise, with ***F***_**i-1**_(*z*) > 0, the three-dimensional equation of motion is used:

12 $$\{\begin{array}{c}-\nabla U{|}_{z={r}_{mb}}\,{\rm{if}}\,{z}_{{\rm{mb}}}\le 0\wedge {{\boldsymbol{F}}}_{{\boldsymbol{i}}-1}(z)\le 0\\ -\nabla U+{{\boldsymbol{F}}}_{{\boldsymbol{G}}}\,{\rm{if}}\,{z}_{{\rm{mb}}} > 0\vee {{\boldsymbol{F}}}_{{\boldsymbol{i}}-1}(z) > 0\end{array}\}$$

In the case that the MB at the end of a solver step is located below the surface level *z*_mb_ < 0, we set *z* to the ground level and the speed along the *z* direction is set to zero. By this approach, in our calculations we prevent the MB from attaining positions below the surface. In the following the code is used to model four individual examples.

## Results and Discussion

**Figure 1:**
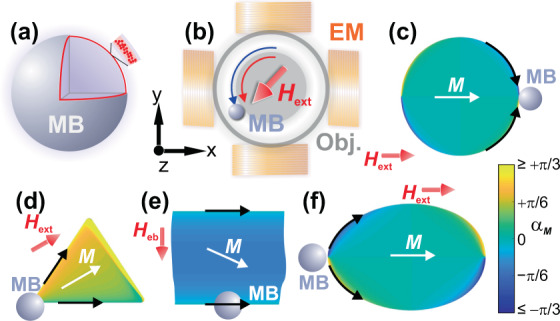
(**a**) Superparamagnetic microbead (MB) and **(b)** basic experimental setup for the manipulation of MBs with an applied magnetic field *H*_ext_ provided by a bi-axial electromagnet (EM). The MB motion is recorded through optical microscopy using an objective lens (Obj.). **(c–f)** Calculated micromagnetic structures of different magnetic structures (see later sects. for details). The angle of magnetization α_m_ is shown.

To show the operation mechanics and range of possible concepts, four different structures are chosen. The material parameters for the structures necessary for the simulations of the micromagnetic structure and the magnetic drag forces are chosen in accordance with corresponding experiments. The used superparamagnetic MB (nanoparticle distribution shown in Fig. 1(a)) are manipulated by a biaxial electromagnet mounted in a microscope (Fig. 1(b)). Examples for all microbead manipulating structures are displayed in Fig. 1(c–f).

### Circular bead motion along microdiscs

The first magnetic parent structure to be investigated is a single circular magnetic element (Fig. 1(c))^18^. In this model structure, the MB is moved around a disk by a rotating magnetic field of fixed amplitude. For faster speeds of magnetic field rotation, in the overcritical regime, looping events were reported, whose dependence on the frequency and external field strength was replicated well by the two-dimensional simulations^18^. However, the experiments indicated that during the looping events the MB potentially lifts from the planar parent structure, a fact that cannot be captured by two-dimensional simulations.

**Figure 2:**
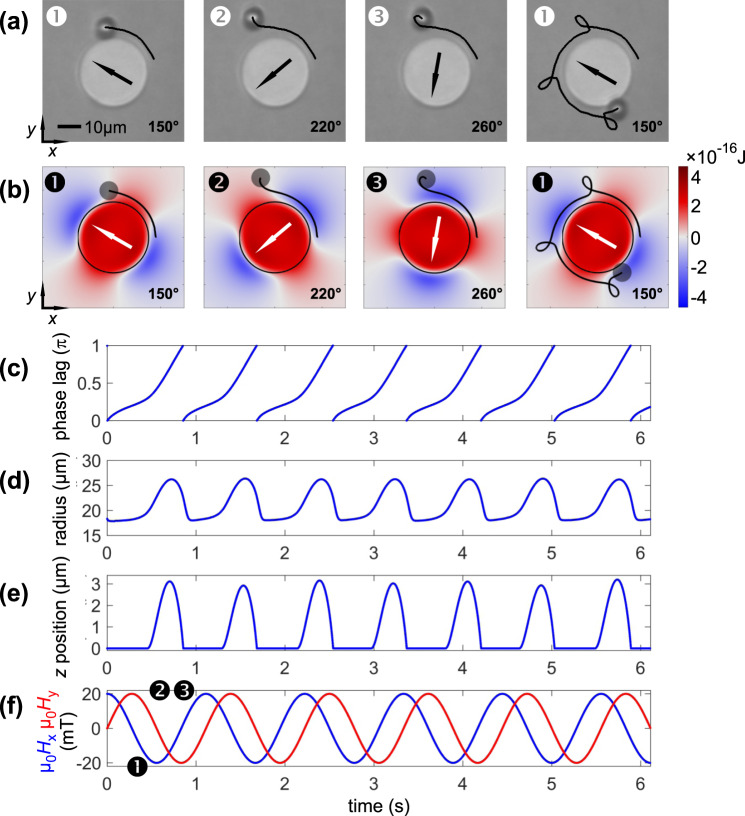
(**a**) Experimental and (**b**) simulated data for a ∅8 µm MB moving around a single circular element with an applied rotating magnetic field of *µ*_*0*_*H*_ext_ = 20 mT and a rotational frequency of *f* = 0.9 Hz. Simulated trajectories are depicted together with the calculated magnetic potential landscape, showing different moments during a single looping event. For 5.5 field periods (equal to approx. 6.1 s) the phase lag (**c**) of the bead relative to the magnetic field orientation, the radial (**d**) and *z* position (**e**) is plotted against time together with (**f**) the corresponding magnetic field components *H*_x_ and *H*_y_ of the rotating field. Magnetic field directions are indicated in (**a**,**b**). The temporal positions of the snapshots in (**a**,**b**) are indicated in (**f**). (Movies of the simulated and experimentally obtained MB motion along the disc are shown in supplementary movie S1 and S2).

The system of interest consists of a single disc Ni_81_Fe_19_ element with a radius of 15 µm and a film thickness of 30 nm. A typical onion-like magnetization state is obtained from the calculations with an applied magnetic field of *µ*_0_*H*_ext_ = 20 mT^18^. The calculated magnetic structure under a magnetic bias field is displayed in Fig. 1(c). The MB trajectory is shown for a rotating field frequency of *f* = 0.9 Hz. Results for the experimental and simulated behaviour of MB motion are displayed in Fig. 2.

Comparing the experimental results (Fig. 2(a)) with the calculated MB trajectories (Fig. 2(b)), a good agreement between experimental and simulated data is attained. The corresponding potential landscape is included for the simulation results. As for the experiments, a looping motion of the MB around the ferromagnetic disc structure is obtained. Analysing the phase lag between the applied field angle and the MB (Fig. 2(c)), the radial (Fig. 2(d)) and the *z* position (Fig. 2(e)) further details of MB motion are revealed. As the phase lag of the MB versus the field angle increases, also the distance of the MB to the structure grows larger. This is in agreement with the experiments (compare Fig. 2(b) to Fig. 2(a)). The simulations show that the onset of disc to MB separation (Fig. 2(d)) is accompanied by a lifting of the MB from the surface (Fig. 2(e)). As the MB lags behind the zero-potential line, the repulsive force leads to a lift along the *z* direction and a higher velocity away from the structure surface. This behaviour is difficult to quantify in experiments, as the varying height information is not accessible by means of optical observation of the spherical MB. On its maximum distance from the parent structure, the MB reaches a height of *z* ≈ 3 µm above the cell surface (with a distance of 10 µm to the edge of the disc structure). The second zero potential line has to be passed to accelerate the MB again towards the structure and to close the loop path.

The looping trajectories of a MB around the circular disc structure and its agreement with the two-dimensional calculations presented in^18^ prove the applicability of the three-dimensional simulations.

### Directional microbead motion across a hexagonal lattice of triangular ferromagnetic elements

The forecast of the trajectory of a MB along a whole array of magnetic elements (Fig. 1(d)), by using periodic boundary conditions, a periodic array of soft magnetic triangles is demonstrated next^25^. For this more complex magnetic structure, alternatingly switching in‐plane applied magnetic fields were used to enable the locomotion of MBs along discrete hexagonally arranged ferromagnetic structures. In the flowless microfluidic environment the MBs were moved along spatial microcorridors for the positioning and sorting of mixed populations MBs. The ways of motion were depending on the field amplitude, direction, and frequency^25^.

**Figure 3:**
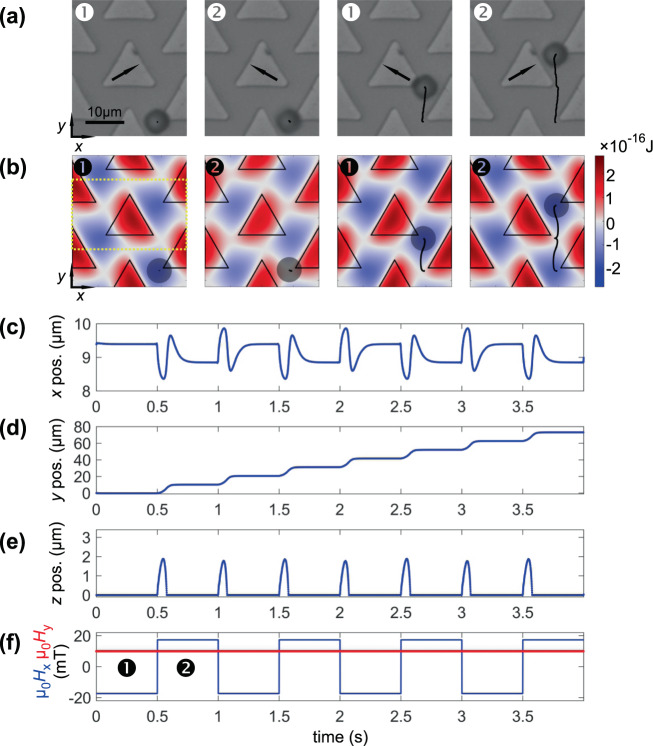
(**a**) Experimental and (**b**) simulated path of MB motion for a ∅8 µm MB moving along a periodic array of triangular elements. The trajectories of motion are indicated. A field of *µ*_*0*_*H*_ext_ = 20 mT is applied with switching frequency of *f* = 1 Hz between an angle of 30° and 150°. The 0° position is along the *x* direction. Corresponding potentials are mapped in (**b**). (**c**–**f**) display the simulated *x*, *y*, and *z* positions, and the magnetic field components *H*_x_ and *H*_y_. Simulation data are shown for 4 field sequences in (**c**–**f**). The field of simulation is indicated in (**b**) on the left. Magnetic field directions are indicated in (**a**). The temporal positions of the snapshots in **(a**,**b**) are indicated in (**f**). (Movies of the simulated and experimentally obtained MB motion along the disc are shown in supplementary movie S3 and S4).

In the shown example in Fig. 3, the upward bead movement with field amplitude *µ*_0_*H*_ext_ = 20 mT and the magnetic field switching between a 30° and a 150° direction are investigated. The alternating magnetic fields are oriented parallel to two mirror axes of the triangles. By this, a movement of the MB in *y* direction between the triangle tips with the respective highest stray fields is achieved. As the MB exhibits repulsion forces after each field switch, a small out-of-plane movement along *z* during the MB’s motion is expected. The shown three-dimensional simulations address this possible behaviour.

The studied triangular elements have a side length of 15 µm and consist of a 50 nm thick Ni_81_Fe_19_ film (see^25^ for details). An exemplary calculated magnetic structure of a single triangular element under a magnetic bias field is displayed in Fig. 1(d). In our computer experiments, the magnetic states alternatingly switch between two different field configurations with a switching frequency of *f* = 1 Hz.

Results of microbead motion from experiments and simulations are displayed in Fig. 3. In experiment and simulation, the MB is moving upward stepwise for the chosen field conditions. The experimental trajectory data indicated in Fig. 3(a) displays a modulated path of movement across the element array. The exact bracket-like path of movement becomes best visible in the simulation data in Fig. 3(b) that is not limited to the camera frame rate of 16 Hz used for the experiments in Fig. 3(a). The small motions in *x* direction, when the MB leaves or reaches a triangle tip are plotted in Fig. 3(c). This detail of motion is not clearly observable in the experiment, due to the discussed lower time resolution in the experiment. The mapped potential landscapes sketched together with the moving MBs allow a deeper understanding of the working principle of the system. The continuous transition of the potentials between the potential of the top and bottom of a unit cell is visualized in Fig. 3(b). Immediately after a field switch, the MB is placed between two different potential minima. The movement upwards is caused by the repulsive force of the bottom triangle edge and the right edge of the neighbouring element, leading to an acceleration in positive *y* direction. This is accompanied by a small lift of *z* = 2 µm above the array surface. The corresponding lateral and height data is displayed in Fig. 3(c–e).

Not only the dynamic MB motion can be reproduced, but from the shown dependences in comparison to the field progression in Fig. 3(f), from the sequent halt of the MB one can suggest a significantly higher critical limit of motion. Higher field switching frequencies seem to be accessible and were also shown experimentally^25^.

### Bead motion across exchange biased microstripes

**Figure 4:**
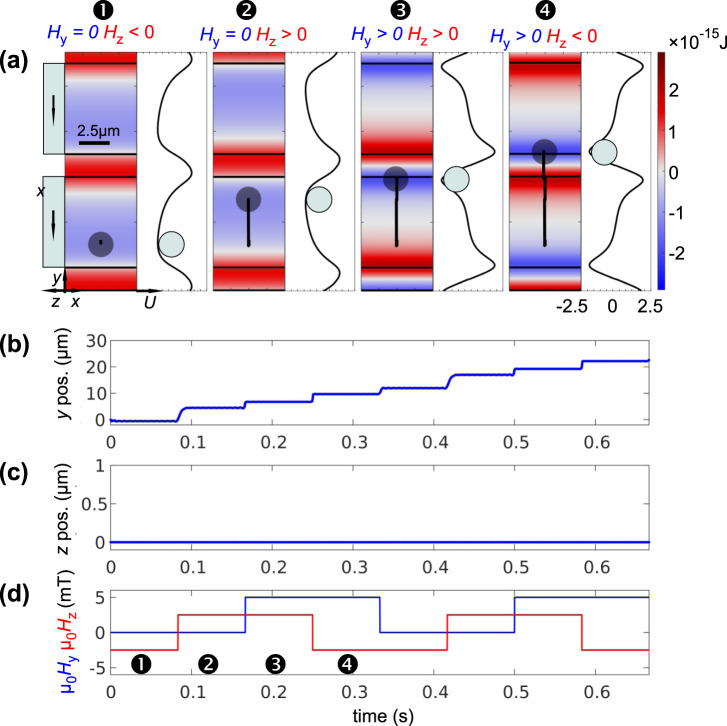
(**a**) Simulated trajectories of ∅2.8 µm microbeads moving over a stripe-patterned exchange biased Ni_81_Fe_19_ structure proposed by Donolato *et al*.^20^. The set directions of magnetization are sketched on the left. Potential landscapes and the spatial dependence of the potential energy in y direction are shown. Movement along (**b**) *y* and (**c**) *z* together with (**d**) the magnetic field sequence. Two field sequences are shown. Applied magnetic field components are indicated in (**a**). The positions of the snapshots in (**a**) are indicated in (**d**). (A movie of the MB motion across the stripes is shown in supplementary movie S5).

To show the wide range of capabilities of MB movement simulations a system based on a complete different concept established by Donolato *et al*.^20^ is selected (Fig. 1(e)). Related concepts of bead movement were applied before^21,24^. Here, the MBs were moved over a repetition of nearly infinite elongated exchange biased ferromagnetic/antiferromagnetic Ni_81_Fe_19_/Ir_20_Mn_80_ microstripes, which are magnetized perpendicular to the stripe axis even without the application of a magnetic field by the induced unidirectional anisotropy. The stripes were 10 µm wide and the ferromagnetic layer thickness was 20 nm. The general quasi two-dimensional arrangement is sketched in Fig. 4(a) (see^20^ for details). The magnetic field is switched between four effectively different configurations alternating the in-plane field component from *µ*_0_*H*_y_ = 0 mT to *µ*_0_*H*_y_ = 5 mT, the latter being antiparallel to the exchange bias, and an additionally alternating out-of-plane field switching from *µ*_0_*H*_z_ = −2.5 mT to *µ*_0_*H*_z_ = 2.5 mT along the *z* direction (Fig. 4(d)). An exemplary calculated magnetic state in a single stripe (*H*_x_ = 0, *H*_y_ = 0) is shown in Fig. 1(e). By the magnetic field sequence an effective 4 step magnetization change occurs due to the phase shift in switching. A magnetic field switching frequency of *f* = 3 Hz is used for the simulations. As a result, the MB travels upwards on top of the microstripes from one stripe edge to another. Note that similar movement is also possible without exchange bias by the application of a bipolar in-plane field *H*_y_.

Simulation results are displayed in Fig. 4. The spatial dependence of the potential along the *y* direction shown in Fig. 4(a) are in agreement to those obtained with static multiphysics simulations^20^. The calculated trajectories shown in Fig. 4(b) display a linear path in positive *y* direction equal to the movement of the MBs in the experiments. Some minor oscillations along the equipotential lines occur, which are due to minor inhomogeneities in the calculated magnetization data. In accordance with the published data, a stepwise motion of the MB along the direction perpendicular to the microstripes becomes visible with the alteration of the magnetic fields (Fig. 4(b)). The calculated almost instantaneous movement of the MB after the switching of the magnetic fields indicates a possible higher achievable effective bead velocity by at least one order of magnitude. No significant motion takes place in the *z* direction perpendicular to the surface (Fig. 4(c)), as the potential keeps the bead to the surface for all magnetization steps.

### Bead motion along a track of oval magnetic structures

Here we predict the MB trajectory for a track consisting of FeCoSiB ferromagnetic oval thin film structures (Fig. 1(f)). (FeCoSiB possesses a higher saturation polarisation than Ni_81_Fe_19_ and therefore provides potential advantages in terms of the generated stray fields). As demonstrated, the concept allows for unidirectional movement of MBs, while applying a rotating magnetic field of constant amplitude. A related concept was demonstrated in^19^. As we show, the movement of beads between these elements is achieved by an asymmetrical potential originated by two different radii of curvature. (Details on the use of such structures for MB transport will be published elsewhere). Nearly equal experimental oval structures, based on FeCoSiB thin films with a nominal thickness *t* = 50 nm, were produced to compare the numerical analysis to experimental results. The use of oval structures for the manipulation of microbeads was first investigated with the simulation program and the results are then verified by experiments.

**Figure 5:**
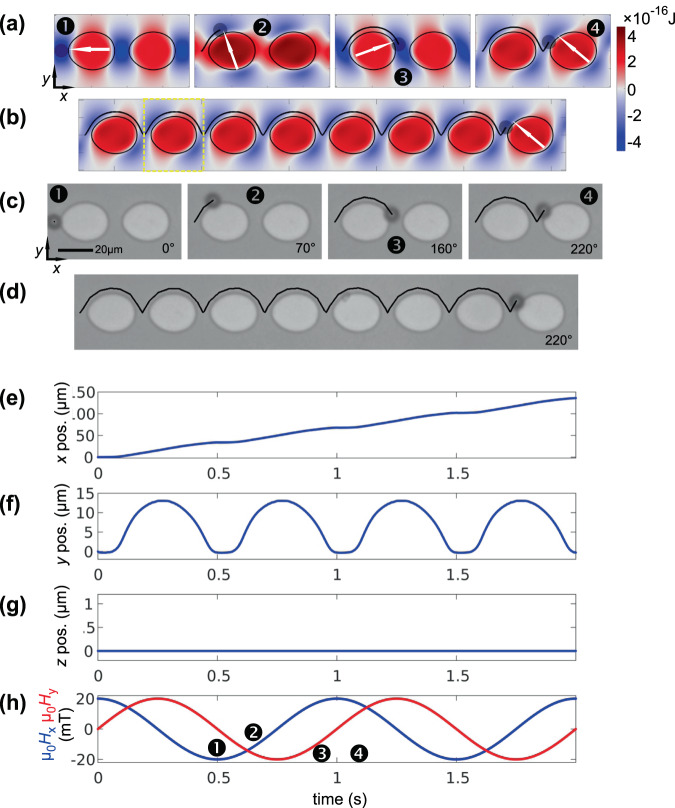
Behaviour of a ∅8 µm MB moving along a string of separated oval structures in an applied rotting field of *µ*_*0*_*H*_ext_ = 20 mT rotating clockwise at *f* = 1.0 Hz. (**a**),(**b**) Snapshots of experimental bead motion and (**d**),(**c**) corresponding simulated MB positions and MB trajectory. The calculated potentials are mapped as a background in (**c**) and (**d**). (**e**–**h**) display the simulated positions and magnetic field components as indicated. Data are depicted for two [(**a**),(**c**)] respectively [(**b**),(**d**)] seven magnetic field rotations. The positions of the snapshots in (**a**,**c**) are specified in (**h**). The unit cell of calculation is indicated in (**b**). (Movies of the simulated and experimentally obtained MB motion along the disc are shown in supplementary movie S6 and S7).

Results of simulations and experiments are displayed in Fig. 5. An exemplary calculated magnetic state with *µ*_0_*H*_x_ = 20 mT is shown in Fig. 1(f). Snapshots of simulated MB motion are shown in Fig. 5(a) and a more extended path of directional MB motion is displayed in Fig. 5(b), respectively for the experiments in Fig. 5(c) and (d). For both, simulations and experiments, the magnetic field rotational frequency was set to *f* = 1 Hz. The starting configurations were set to a field angle of zero degree with the MB in the overlapping potential minimum between two neighbouring elements. In the horizontally aligned magnetic field, with the MB situated between the oval structures, the MB is located towards the element edge with the smaller radius of curvature. This is a result of the sharper potential well and thus results in a higher attractive force from the tip as compared to the base of the ovals. By using periodic boundary conditions, a continuous transition between the potential values of the left and right edge is achieved, which can be observed by concatenating the potential landscapes with themselves.

As the rotational frequency of the applied magnetic fields used during the experiments and simulations is low, the MB is always bound to the magnetic structures. For none of the field configurations a movement of the MB along the *z* dimension takes place (z ≈ 0). The corresponding absence of *z* motion of the MB is seen from the time dependence of the MB position (Fig. 5(g)). Moreover, from Fig. 5(f) the MB rests for approx. 120 ms at the position between two oval elements, as their potentials start to overlap also for field angles of 20° and 160°, respectively. All the predicted dependencies are also found in the experiments. The simulation data accurately predict the experimental results, demonstrating the possibility of proving new concepts by the presented numerical framework.

## Conclusions

The movement behaviours of MBs on soft magnetic parent structures for lab-on-chip applications was estimated by simulation and compared to experiments. Small discrepancies in the MB behaviour are within the error margin of the experiment for the selected parameters. This includes errors in terms of the applied magnetic field direction and amplitude, the detection accuracy of the MB position detection, as well as structural errors like lithographic errors. Within these limits, a high consistency of simulation and experimental data is established. Using periodic boundaries, the behaviour along extended and with more complicated structures can be calculated. Simulating and analysing the MB movement in three dimensions leads to insights in MB motion not apparent from experiments.

Using the presented method, the magnetic manipulation of MBs can be predicted with high accuracy. The demonstrated numerical framework for computer experiments is a valuable tool for designing, understanding, and optimizing new potential magnetic parent structures for MB manipulation. It provides a corner stone for the design of future experiments and lab-on-chip applications.

## Methods

### Magnetic structures

The used ferromagnetic structures consist of Ni_81_Fe_19_ (saturation polarization *J*_s_ = 1 T, exchange constant *A*_ex_ = 1.3 × 10^−11^ J/m)^39^ or amorphous (Fe_90_Co_10_)_78_Si_12_B_10_ films (*J*_s_ = 1.5 T^40^, *A*_ex_ = 1.5 × 10^−11^ J/m). The films were prepared by sputter deposition on oxidized silicon wafers or on top of glass substrates and protected by up to 5 nm thick Ta or TaN capping layers. The magnetic element arrays were patterned by photolithography followed by ion beam etching process.

### Superparamagnetic microbeads

The used 8 µm diameter polystyrene micromer-M MBs were obtained by micromod Partikeltechnologie GmbH^41^. The MBs contain monodispersed superparamagnetic magnetite nanoparticles embedded in the outer surface of the MB, which are encapsulated by an outer polymer coating (Fig. 1(a)).

### Microfluidic cell

Microfluidic cells of about 10 mm × 10 mm × 0.5 ± 0.1 mm were fabricated stacking paraffin–polyethylene sealing film rings (Parafilm M) on the Si substrates and sealed by a cover glass. Movement of the MBs is achieved using an aqueous solution with a MB concentration of 5×10^−6^ mg/ml and a content of 0.01% to 0.03% Triton X-100. The measured water temperature was 22 °C during the experiments. The same value was applied for the calculation of the viscosity of water.

### Observation of microbead motion

An optical microscope equipped with a digital CCD camera and a biaxial electromagnet was used to move and track the MBs’ motion (Fig. 1(b)). Magnetic field amplitude and angle was recorded for all image frames. ImageJ^42^ was used to determine the trajectory of MB movement with the application of time-varying magnetic fields.

## Simulations

### Circular bead motion along microdiscs

Micromagnetic simulations are performed for a single disc Ni_81_Fe_19_ element with a radius of 15 µm and a film thickness of 30 nm. A mesh grid of 1700 × 1700 × 3 with a cell size of 18 nm × 18 nm ×10 nm is used. From the calculated magnetization state, which is obtained by relaxing the disc magnetization out of saturation with an applied field of *µ*_0_*H*_ext_ = 20 mT^18^, the magnetic potential landscape is calculated. Potentials for other magnetic field angles are obtained by a matrix rotation operation without recalculating the magnetic state. To reduce calculation time, the magnetization grids are averaged by a factor of 10. The potential landscapes are then calculated on a 200 × 200 grid for 41 different *z* layers, ranging between the MB radius (ground level) and three times the MB radius. A single additional layer 200 nm beneath the ground level is added for spatial fitting along the *z* direction. A time resolution of *t*_res_ = 0.7 ms is used for the calculations of the MB trajectories. For this a time fit through the potential maps is performed with a Fourier series of order *N* = 30. The spatial dependence of the potential is fitted using submatrices with a size of 3 × 3 × 3. We estimate the friction coefficient based on the radial distance of the looping events of the MBs. A best fit to experimental results is achieved for a constant F_c_ = 0.03, while the experimental spread in looping distances of about 2% would allow for an error margin of the friction coefficient of approximately 100%. The shown MB susceptibility, used as a scaling factor, is then set in a way that the number of looping events is equal to the number observed in the experiments under same conditions. The used susceptibility of χ = 0.0256 for the three-dimensional simulations is comparable to the values estimated in the previous two-dimensional system^18^. This value of susceptibility is consisting with the occurrence of the peak of critical frequency for a batch of microbeads (see Supplementary Figure S1 for a the distribution of frequencies for one batch of microbeads). Also other effects like discrepancies in the bead size and shape, the magnetic content, and surface properties might contribute to the distribution of critical frequencies. The stated susceptibility value was used for all of the further simulations, using the same type of MBs.

### Directional bead motion across a hexagonal lattice of triangular ferromagnetic elements

The simulation of the trajectory of a MB along a whole array of triangular magnetic elements is addressed using periodic boundary conditions. An field amplitude of *µ*_0_*H*_ext_ = 20 mT switching between a 30° and a 150° direction are used for the simulations. For the micromagnetic simulations a grid mesh of 2048 × 1024 × 4 with a cell size of 17.8 nm × 20.5 nm × 12.5 nm is used, containing a full triangle in the centre and a quarter of a triangle in each corner as a unit cell for the hexagonal arrangement (indicated in Fig. 3(b), left). An exemplary calculated magnetic structure of a single triangular element under a magnetic bias field is displayed in Fig. 1(d). For the micromagnetic simulations periodic boundary conditions are applied as the magnetic states for the two different field configurations, switching with *f* = 1 Hz, are determined. The magnetization grid is averaged by a factor of four before the potential calculation, where also onefold periodic boundary conditions are applied. The potential landscapes are resolved to a 100 × 100 grid and 22 layers in z direction up to a height of 12 µm. As in the time dependence the magnetic potential in every point for this system switches between two states, 100 times the potential for a magnetic field angle of 30° is concatenated to 100 times the potential for a field angle of 150°. It is further fitted by a Fourier algorithm of order *N* = 100 to obtain the appropriate square wave magnetic field function. Subsequently a spatial fit with a submatrix size of 3 × 3 × 3 is performed.

### Bead motion across exchange biased microstripes

For the simulations of the ferromagnetic/antiferromagnetic Ni_81_Fe_19_/Ir_20_Mn_80_ microstripes we assume the magnetization of the 10 µm wide and 20 nm thick stripes to be constant over their length (*l* = 6 mm). The magnetic field is switched between four effectively different configurations alternating the in-plane field component from *µ*_0_*H*_y_ = 0 mT to *µ*_0_*H*_y_ = 5 mT and an additionally alternating out-of-plane field switching from *µ*_0_*H*_z_ = −2.5 mT to *µ*_0_*H*_z_ = 2.5 mT along the *z* direction. For the quasi two-dimensional structure, the micromagnetic state is calculated for only a small section of approximately 100 nm in width, resulting in a grid mesh of 8 × 1024 × 8 with cell sizes of 12.2 nm × 12.2 nm × 2.5 nm with 300-fold periodic boundary conditions along the *x* direction and onefold periodic boundary conditions applied the *y* direction. Exchange bias is introduced in the simulations by adding an exchange field of *µ*_0_*H*_eb_ = −5.5 mT to the applied magnetic field for the calculation of the distribution of magnetization. The added external bias field is then neglected for the computing of the potential and the bead trajectory to avoid artificial effects on the magnetization of the MB. For the potential maps also 300-fold and onefold periodic boundary conditions are applied along *x* and *y* direction and the magnetization grid is averaged by factor of four. The potential grid mesh is 8 × 100 and is calculated for 16 heights ranging up to 4.2 µm. In our simulations, we adopt The same susceptibility as for our larger MBs is used for the calculations. The four different obtained potentials are concatenated similar to the triangles and then fitted by a Fourier series of order *N* = 50. The spatial fit is done by a submatrix of 3 × 3 × 3. In accordance with ref. ^20^ a switching frequency of *f* = 3 Hz is used for the simulations.

### Bead motion along a track of oval magnetic structures

The micromagnetic state of a 26 µm × 21 µm × 50 nm large FeCoSiB oval structure is calculated with cell sizes of 16.6 nm × 20 nm × 50 nm, corresponding a mesh grid of 2048 × 2048 × 1. As the asymmetric ferromagnetic structures lack in point symmetry the magnetization states for 180 angles between 0° and 358° of the applied clockwise rotating magnetic field (*µ*_0_*H*_ext_ = 20 mT) are calculated. An exemplary calculated magnetic structure of a single oval element is shown in Fig. 1(f). For the calculations of the magnetic potential landscapes, the magnetization grids are averaged down by a factor of 8. The same value of susceptibility χ as in the previous experiments is assumed for the MB. The potential landscape is calculated with onefold periodic boundary conditions and a 125 × 125 in-plane resolution. Potentials are evaluated for 6 different *z* layers in the range of half to three times the MB radius. To obtain an effective higher time resolution, the potential sequence is fitted by a Fourier algorithm of order *N* = 30. The submatrix size for the spatial fit is selected to 3 × 3 × 3. Nearly equal experimental oval structures, based on FeCoSiB thin films with a nominal thickness *t* = 50 nm, were produced by sputtering and subsequent patterning using photolithography and ion beam etching to compare the numerical analysis to experimental results.

## Supplementary information

Supplementary Information.

Supplementary Movie 1.

Supplementary Movie 2.

Supplementary Movie 3.

Supplementary Movie 4.

Supplementary Movie 5.

Supplementary Movie 6.

Supplementary Movie 7.

## Data Availability

The datasets generated during the current study and the simulation code are available from the corresponding author on reasonable request.
