# Supplementary material for: Evaluating and forecasting movement patterns of magnetically driven microbeads in complex geometries
Source: Sci Rep. 2020 May 29;10:8761. doi: 10.1038/s41598-020-65380-8 (PMC7260204; doi:10.1038/s41598-020-65380-8)
Supplement: Supplementary file 1 — Supplementary Information. [file 41598_2020_65380_MOESM1_ESM.docx]

Supplementary information for

**Evaluating and forecasting movement patterns of magnetically driven microbeads in complex geometries**

**Finn Klingbeil, Findan Block, Umer Sajjad, Rasmus B. Holländer, Sughosh Deshpande, Jeffrey McCord**

**Fig. S1** Distribution of critical frequency f_crit_ of microbead motion around a circular disk. The microbead diameter is 8 µm. The applied rotating magnetic field is µ_0_*H*_ext_ = 20 mT.
